# Potentially functional variants of autophagy‐related genes are associated with the efficacy and toxicity of radiotherapy in patients with nasopharyngeal carcinoma

**DOI:** 10.1002/mgg3.1030

**Published:** 2019-11-06

**Authors:** Zhiguang Yang, Zhaoyu Liu

**Affiliations:** ^1^ Department of Nuclear medicine Shengjing Hospital Affiliated to China Medical University Shenyang China; ^2^ Department of Radiology Shengjing Hospital Affiliated to China Medical University Shenyang China

**Keywords:** autophagy‐related genes, genetic, nasopharyngeal carcinoma, radiotherapy

## Abstract

**Background:**

Nasopharyngeal carcinoma (NPC) is one of the major invasive malignant neoplasms of head and neck, while radiotherapy is the primary therapy for NPC. Genetic variants could affect the efficacy and toxicities of radiotherapy in NPC patients.

**Methods:**

In the current study, we aimed to investigate 10 potentially functional SNPs of autophagy‐related genes (*ATG*) with the efficacy and toxicity of radiotherapy in 468 NPC patients.

**Results:**

We found *ATG10* rs10514231, rs1864183, and rs4703533 were significantly associated with worse efficacy of radiotherapy at both at the primary tumor and lymph node, while *ATG16L2* rs10898880 was significantly associated with better efficacy of radiotherapy at both primary tumor and lymph node. Besides, we also found *ATG10* rs10514231 and *ATG16L2* rs10898880 were significantly associated with the occurrence of grade 3–4 oral mucositis (allelic model, for rs10514231: OR = 1.95, 95% CIs = 1.31–2.9, *p* = .001; for rs10898880: OR = 1.56, 95% CIs = 1.19–2.04, *p* = .001) and grade 3–4 myelosuppression (allelic model, for rs10514231: OR = 2.08, 95% CIs = 1.39–3.09, *p* < .001; for rs10898880: OR = 1.51, 95% CIs = 1.1–2.06, *p* = .010).

**Conclusions:**

This should be the first report identifying *ATG10* rs10514231, rs1864183, rs4703533, and *ATG16L2* rs10898880 could contribute to the efficacy and toxicity of radiotherapy in NPC patients. Further investigation of the underlying molecular mechanisms and prospective clinical trials in NPC patients are needed to validate our results.

## Graphical abstract

This should be the first report identifying ATG10 rs10514231, rs1864183, rs4703533, and ATG16L2 rs10898880 could contribute to the efficacy and toxicities of radiotherapy in NPC patients.

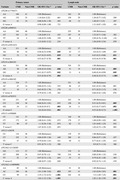

## 1 INTRODUCTION

Nasopharyngeal carcinoma (NPC) was one of the major invasive malignant neoplasms of the head and neck (Clifford, 1970; Huang, 1990; Yu, Ho, Henderson, & Armstrong, 1985). It was especially prevalent in China, southeastern Asia, the natives of the Artic region, and the Arabs of North Africa and parts of the Middle East (Kamal & Samarrai, 1999; Yu & Yuan, 2002). In Indonesia, the mean prevalence was 6.2/100,000, with 13,000 yearly new NPC cases (Adham et al., 2012). While in southern China it is much greater, with annual rates between 15 and 30 NPC cases per 100,000 (Kamran, Riaz, & Lee, 2015). Radiotherapy alone or chemoradiotherapy, is an important component of the primary therapy of NPC for its highly radio‐sensitivity (Miao et al., 2019; Zhan, Zhang, Wei, Fu, & Zheng, 2019). However, predictors of the efficacy and toxicity response to radiotherapy of NPC have not been yet fully identified (Chen et al., 2019; Kamran et al., 2015; Miao et al., 2019).

The discovery of suitable biomarkers is needed to predict efficacy and toxicity of radiotherapy in patients with NPC. Recently, single nucleotide polymorphisms (SNPs) of candidate genes have been to be associated with the outcomes and toxicity in patients accepting radiotherapy of many cancers, including lung cancer, NPC, prostate cancer, breast cancer, oropharyngeal cancer, thyroid cancer, and so on (Kerns et al., 2019; Lewin et al., 2019; Liu et al., 2018; Tao et al., 2018; Wang et al., 2017; Wen et al., 2018). Studies showed that autophagy played an important role in various stages of cancer development, progression, radio‐sensitivity and toxicity, including NPC (Liang et al., 2018; Lin et al., 2014; Qin et al., 2013; Wen et al., 2018; K. Xie et al., 2016; Yang et al., 2018; Yuan et al., 2017; Zhu et al., 2018). Autophagy could selectively target dysfunctional organelles, intracellular microbes, and pathogenic proteins, and deficiencies in these processes might lead to occurrence of cancers (Levine & Kroemer, 2019). During this process, autophagy‐related genes (*ATG*) play an essential role in autophagy, and directly or indirectly accelerate cancer development and progression (Levine & Kroemer, 2019; Tsuboyama et al., 2016). The *ATG* family is a big family, and only a small part of the family members are currently known in humans (Klionsky, 2007). Some potentially functional variants of *ATGs* have been identified to be associated with the development, progression, radio‐sensitivity and toxicity of other cancers, like lung cancer, hepatocellular carcinoma, prostate cancer, bladder cancer, breast cancer, and so on (Budak Diler & Aybuga, 2018; Li et al., 2019; Nikseresht et al., 2018; Zhou et al., 2017). Inspired by these findings, the present study was conducted to establish the relationships, if any, between potentially functional variants of *ATGs* and the efficacy of radiotherapy, as well as radiation‐induced toxicity reaction in NPC patients.

## 2 PATIENTS AND METHODS

### 2.1 Study populations

The current study totally recruited 468 pathological diagnosed NPC patients treated with radiotherapy. The inclusion criteria was a first‐time diagnosis of NPC, no prior treatment of anticancer therapies, no severe disorders of lung, heart, liver, pancreas, or kidney diseases. At recruitment, each participant or family members signed the informed consent form and a 5 ml of blood sample from the patients was collected. Genetic DNA of all patients was extracted using Wizard Genomic DNA Purification Kit (Promega), and stored at −80°C for further evaluation. Questionnaires on patient demographics were collected prior to treatment. The present study's protocol was approved by the ethics committee of the Shengjing Hospital Affiliated to China Medical University.

### 2.2 Treatment efficacies and toxic reactions

All the patients were treated with intensity modulated radiation‐therapy (IMRT), with a tumoricidal radiation dose of 66–70 Gy in 30–33 fractions for nasopharyngeal primary focus and the positive lymph nodes. All the patients underwent fluorodeoxyglucose‐positron emission tomography/computed tomography (FDG‐PET/CT) after treatment. Treatment efficacies at the primary tumor and lymph node were evaluated in line with the Response Criteria in Solid Tumors (PERCIST), which defined treatment efficacy as complete metabolic response (CMR). Radiation‐induced toxic reactions, including.

dermatitis, oral mucositis and myelosuppression, were evaluated according to the radiation toxicity grading criteria of the Radiation Therapy Oncology Group or European Organization for Research and Efficacy of Cancer (RTOG/EORTC). Patients were defined as “non‐sensitive or mildly radiosensitive” group (grade 0–2) and “highly radiosensitive” group (grade 3–4).

### 2.3 Selection of SNPs and genotyping

The selection of candidate SNPs were mainly based on study previously published by Wen et al. (2018). Eight of the nine functional SNPs [*ATG2B* rs17784271 (3ʹUTR) and rs4900321 (3ʹUTR); *ATG10* rs10514231 (intron 2), and rs4703533 (the promoter region); *ATG12* rs26538 (the promoter region) and rs1058600 (3ʹUTR); *ATG16L2* rs1126205 (the promoter region) and rs10898880 (the promoter region)], were included (MAF of rs6884232 in Chinese was 0). We also included two widely reported SNPs in *ATG10* gene, rs1864182 and rs1864183. This means totally 10 SNPs were included in this study. The genotyping was performed using the TaqMan methodology and read with the Sequence Detection Software on an ABI‐Prism 7,900 instrument according to the manufacturer's instructions (Applied Biosystems, Foster City, CA).

### 2.4 Statistical analysis

All statistical tests were two‐sided and a *p* value of .05 was considered significant, and all analyses were performed using SAS software version 9.2 (SAS Institute). Univariate logistic regression was performed to determine the association of the 10 SNPs with the efficacy at the primary tumor and lymph node, as well as the radiation‐induced toxicity reaction in NPC patients adjustment for age, gender, BMI, smoking, drinking, family history of cancer, EBV‐DNA, chemotherapy, and TNM stage.

## 3 RESULTS

### 3.1 Population characteristics and clinical outcomes

The baseline demographics and clinical profiles are presented in Table 1. Totally 468 histopathological confirmed NPC cases, with a mean age of 49 (*SD* = 11), 319 male cases (68.2%), and a mean BMI of 23.1 (*SD* = 4.3), were included in this study. Among them, 128 (27.3%) were smokers, while 153 (32.7%) were drinkers. Plasma level of Epstein Barr virus (EBV) was detectable in 330 (70.5%) cases. Eighty‐two (17.5%) cases had family history of cancer, and 349 (74.6%) accepted chemotherapy meanwhile. The TNM stage distribution of all NPC patients were 61 (13.0%) for I or II, 284 (60.7%) for III, and 123 (26.3%) for IV, respectively. Overall, there were 95 (20.5%) and 80 (17.1%) patients who did not get CMR after radiotherapy at their primary tumors and lymph nodes, respectively. For the toxic reactions, 55 (11.8%), 242 (51.7%), 118 (25.2%) patients experienced grade 3–4 acute radiation‐induced dermatitis, oral mucositis, and myelosuppression, respectively.

**Table 1:** NPC patient demographics and clinical characteristics

Characteristics	Patients (%)/values (*N* = 468)
Age	49 ± 11
Gender	
Male	319 (68.2%)
Female	149 (31.8%)
BMI	23.1 ± 4.3
Smoking	
Yes	128 (27.3%)
No	340 (72.7%)
Drinking	
Yes	153 (32.7%)
No	315 (67.3%)
EBV‐DNA	
Positive	330 (70.5%)
Negative	138 (29.5%)
Family history of cancer	
Yes	82 (17.5%)
No	386 (82.5%)
Chemotherapy	
Yes	349 (74.6%)
No	119 (25.4%)
TNM stage	
I, II	61 (13.0%)
III	284 (60.7%)
IV	123 (26.3%)
Non‐CMR after radiotherapy	
Primary tumor	95 (20.5%)
Lymph node	80 (17.1%)
Grade 3–4 radiation‐induced toxic reactions	
Dermatitis	55 (11.8%)
Oral mucositis	242 (51.7%)
Myelosuppression	118 (25.2%)

### 3.2 Associations between candidate SNPs and the efficacy of radiotherapy

Table 2 presents the associations between candidate SNPs and the efficacy of radiotherapy at the primary tumor and lymph node in NPC patients. We found *ATG10* rs10514231, rs1864183, and rs4703533, were significantly associated with worse efficacy of radiotherapy at both at the primary tumor (allelic model, for rs10514231: OR = 0.53, 95% CIs = 0.37–0.78, *p* = .001; for rs1864183: OR = 0.53, 95% CIs = 0.36–0.79, *p* = .002; for rs4703533: OR = 0.60, 95% CIs = 0.44–0.81, *p* = .001) and lymph node (allelic model, for rs10514231: OR = 0.52, 95% CIs = 0.35–0.78, *p* = .001; for rs1864183: OR = 0.48, 95% CIs = 0.32–0.73, *p* < .001; for rs4703533: OR = 0.53, 95% CIs = 0.38–0.73, *p* < .001). While *ATG16L2* rs10898880 was significantly associated with better efficacy of radiotherapy at both at the primary tumor (allelic model: OR = 1.84, 95% CIs = 1.32–2.56, *p* < .001) and lymph node (allelic model: OR = 1.82, 95% CIs = 1.28–2.59, *p* = .001).

**Table 2:** Association between candidate SNPs and the efficacy of radiotherapy at the primary tumor and lymph node in NPC patients

Variants	Primary tumor				Lymph node			
	CMR	Non‐CMR	OR (95% CIs) *	*p* value	CMR	Non‐CMR	OR (95% CIs) *	*p* value
*ATG2B* rs17784271								
AA	160	41	1.00 (Reference)		162	39	1.00 (Reference)	
AG	152	35	1.16 (0.6–2.22)	.661	158	29	1.36 (0.77–2.42)	.290
GG	61	19	0.88 (0.56–1.39)	.583	68	12	1.48 (0.7–3.15)	.307
G versus A			0.98 (0.89–1.08)	.721			1.31 (0.87–1.95)	.194
*ATG2B* rs4900321								
AA	246	68	1.00 (Reference)		255	59	1.00 (Reference)	
AT	103	23	1.26 (0.69–2.3)	.452	107	19	1.32 (0.71–2.46)	.382
TT	24	4	1.68 (0.55–5.09)	.359	26	2	2.99 (0.76–11.69)	.116
T versus A			1.33 (0.83–2.12)	.234			1.56 (0.95–2.54)	.078
*ATG10* rs10514231								
AA	311	68	1.00 (Reference)		322	57	1.00 (Reference)	
AG	40	16	0.56 (0.32–0.99)	**.045**	43	13	0.6 (0.33–1.09)	.095
GG	22	11	0.47 (0.23–0.93)	**.029**	23	10	0.43 (0.21–0.88)	**.021**
G versus A			0.53 (0.37–0.78)	**.001**			0.52 (0.35–0.78)	**.001**
*ATG10* rs1864183								
AA	311	68	1.00 (Reference)		324	55	1.00 (Reference)	
AG	50	20	0.56 (0.34–0.94)	**.028**	51	19	0.47 (0.28–0.8)	**.005**
GG	12	7	0.41 (0.17–0.97)	**.042**	13	6	0.40 (0.16–0.99)	**.049**
G versus A			0.53 (0.36–0.79)	**.002**			0.48 (0.32–0.73)	**<.001**
*ATG10* rs1864182								
AA	316	77	1.00 (Reference)		331	62	1.00 (Reference)	
AC	47	14	0.86 (0.53–1.41)	.552	46	15	0.61 (0.34–1.06)	.081
CC	10	4	0.63 (0.22–1.76)	.376	11	3	0.71 (0.23–2.13)	.538
C versus A			0.79 (0.52–1.19)	.261			0.66 (0.42–1.04)	.076
*ATG10* rs4703533								
CC	231	45	1.00 (Reference)		242	34	1.00 (Reference)	
CG	118	36	0.66 (0.43–1.01)	.053	120	34	0.51 (0.32–0.81)	**.005**
GG	24	13	0.36 (0.19–0.7)	**.003**	26	11	0.33 (0.17–0.67)	**.002**
G versus C			0.6 (0.44–0.81)	**.001**			0.53 (0.38–0.73)	**<.001**
*ATG12* rs1058600								
CC	141	37	1.00 (Reference)		148	30	1.00 (Reference)	
CT	172	43	1.08 (0.41–2.82)	.875	178	37	1.00 (0.99–1.02)	.882
TT	60	15	1.1 (0.36–3.38)	.874	62	13	1.01 (0.82–1.25)	.932
T versus C			1.07 (0.52–2.22)	.853			1.02 (0.75–1.39)	.905
*ATG12* rs26538								
CC	134	30	1.00 (Reference)		139	25	1.00 (Reference)	
CT	192	51	0.86 (0.59–1.26)	.449	200	43	0.86 (0.57–1.29)	.457
TT	47	14	0.79 (0.44–1.41)	.421	49	12	0.77 (0.41–1.45)	.414
T versus C			0.91 (0.73–1.13)	.388			0.9 (0.71–1.14)	.386
*ATG16L2* rs1126205								
GG	113	27	1.00 (Reference)		119	21	1.00 (Reference)	
GT	208	54	0.97 (0.78–1.2)	.780	216	46	0.88 (0.58–1.32)	.530
TT	52	14	0.97 (0.72–1.32)	.844	53	13	0.79 (0.42–1.48)	.465
T versus G			1.00 (0.97–1.03)	.820			0.92 (0.74–1.15)	.470
*ATG16L2* rs10898880								
AA	85	36	1.00 (Reference)		91	30	1.00 (Reference)	
AC	189	46	1.81 (1.08–3.02)	**.023**	195	41	1.63 (0.94–2.83)	**.081**
CC	99	13	3.35 (1.72–6.53)	**<.001**	102	10	3.5 (1.69–7.25)	**.001**
C versus A			1.84 (1.32–2.56)	**<.001**			1.82 (1.28–2.59)	**.001**

Note

*p* value in bold means statistically significant.

* Age, gender, BMI, smoking, drinking, family history of cancer, EBV‐DNA, chemotherapy, and TNM stage

### 3.3 Associations between the candidate SNPs and grade 3–4 radiation‐induced toxic reactions

Tables 3 and 4 presents the associations between the candidate SNPs and grade 3–4 radiation‐induced oral mucositis and myelosuppression, respectively. We found *ATG10* rs10514231 and *ATG16L2* rs10898880 were significantly associated with the occurrence of grade 3–4 oral mucositis (allelic model, for rs10514231: OR = 1.95, 95% CIs = 1.31–2.9, *p* = .001; for rs10898880: OR = 1.56, 95% CIs = 1.19–2.04, *p* = .001) and grade 3–4 myelosuppression (allelic model, for rs10514231: OR = 2.08, 95% CIs = 1.39–3.09, *p* < .001; for rs10898880: OR = 1.51, 95% CIs = 1.1–2.06, *p* = .010). We did not find significant associations for grade 3–4 radiation‐induced dermatitis, due to the small sample size.

**Table 3:** Association between candidate SNPs and Grade 3–4 radiation‐induced oral mucositis

Variants	Grade 3–4	Grade 0–2	OR (95% CIs)*	*p* value
*ATG2B* rs17784271				
AA	98	103	1.00 (Reference)	
AG	100	87	1.26 (0.79–1.99)	.329
GG	44	36	1.36 (0.77–2.4)	.295
G versus A			1.23 (0.89–1.68)	.205
*ATG2B* rs4900321				
AA	158	156	1.00 (Reference)	
AT	67	59	1.16 (0.67–1.98)	.600
TT	17	11	1.57 (0.69–3.56)	.280
T versus A			1.25 (0.86–1.82)	.252
*ATG10* rs10514231				
CC	185	194	1.00 (Reference)	
CT	35	21	1.8 (1.01–3.23)	**.047**
TT	22	11	2.23 (1.06–4.69)	**.035**
T versus C			1.95 (1.31–2.9)	**.001**
*ATG10* rs1864183				
AA	197	182	1.00 (Reference)	
AG	35	35	0.96 (0.74–1.24)	.733
GG	10	9	1.1 (0.25–4.86)	.898
G versus A			1.01 (0.86–1.19)	.887
*ATG10* rs1864182				
AA	204	189	1.00 (Reference)	
AC	31	30	1 (0.98–1.02)	.886
CC	7	7	0.96 (0.57–1.62)	.875
C versus A			0.99 (0.91–1.07)	.819
*ATG10* rs4703533				
CC	143	133	1.00 (Reference)	
CG	82	72	1.1 (0.57–2.12)	.785
GG	17	20	0.81 (0.46–1.4)	.442
G versus C			0.98 (0.89–1.08)	.681
*ATG12* rs1058600				
CC	92	86	1.00 (Reference)	
CT	111	104	1.03 (0.17–6.19)	.972
TT	39	36	1.05 (0.15–7.42)	.957
T versus C			1.04 (0.08–13.08)	.973
*ATG12* rs26538				
CC	85	79	1.00 (Reference)	
CT	125	118	1.02 (0.74–1.41)	.913
TT	32	29	1.07 (0.28–4.09)	.922
T versus C			1.04 (0.08–13.08)	.973
*ATG16L2* rs1126205				
GG	72	68	1.00 (Reference)	
GT	136	126	1.07 (0.38–2.95)	.902
TT	34	32	1.07 (0.25–4.58)	.932
T versus G			1.05 (0.38–2.91)	.920
*ATG16L2* rs10898880				
AA	47	74	1.00 (Reference)	
AC	129	106	1.99 (1.27–3.12)	**.003**
CC	66	46	2.35 (1.4–3.95)	**.001**
C versus A			1.56 (1.19–2.04)	**.001**

Note

*p* value in bold means statistically significant.

* Age, gender, BMI, smoking, drinking, family history of cancer, EBV‐DNA, chemotherapy, and TNM stage.

**Table 4:** Association between candidate SNPs and Grade 3–4 radiation‐induced myelosuppression

Variants	Grade 3–4	Grade 0–2	OR (95% CIs)*	*p* value
*ATG2B* rs17784271				
AA	47	154	1.00 (Reference)	
AG	49	138	1.21 (0.7–2.1)	.500
GG	22	58	1.3 (0.67–2.52)	.429
G versus A			1.19 (0.82–1.73)	.353
*ATG2B* rs4900321				
AA	76	238	1.00 (Reference)	
AT	33	93	1.15 (0.61–2.16)	.665
TT	9	19	1.53 (0.64–3.66)	.336
T versus A			1.24 (0.81–1.89)	.321
*ATG10* rs10514231				
CC	85	294	1.00 (Reference)	
CT	20	36	1.99 (1.1–3.61)	**.024**
TT	13	20	2.37 (1.15–4.88)	**.020**
T versus C			2.08 (1.39–3.09)	**<.001**
*ATG10* rs1864183				
AA	95	284	1.00 (Reference)	
AG	18	52	1.07 (0.3–3.81)	.914
GG	5	14	1.13 (0.26–4.87)	.866
G versus A			1.09 (0.49–2.42)	.831
*ATG10* rs1864182				
AA	99	294	1.00 (Reference)	
AC	15	46	1.01 (0.8–1.28)	.926
CC	4	10	1.23 (0.31–4.93)	.769
C versus A			1.08 (0.41–2.84)	.876
*ATG10* rs4703533				
CC	73	203	1.00 (Reference)	
CG	37	117	0.91 (0.67–1.24)	.553
GG	8	29	0.79 (0.4–1.54)	.485
G versus C			0.89 (0.7–1.14)	.376
*ATG12* rs1058600				
CC	45	133	1.00 (Reference)	
CT	53	161	1.01 (0.84–1.22)	.902
TT	20	56	1.1 (0.4–3.01)	.856
T versus C			1.06 (0.41–2.73)	.908
*ATG12* rs26538				
CC	42	122	1.00 (Reference)	
CT	61	182	1.01 (0.89–1.14)	.891
TT	15	46	0.99 (0.84–1.16)	.874
T versus C			1.01 (0.86–1.2)	.866
*ATG16L2* rs1126205				
GG	39	101	1.00 (Reference)	
GT	64	198	0.87 (0.62–1.23)	.440
TT	15	51	0.8 (0.46–1.4)	.436
T versus G			0.92 (0.76–1.12)	.413
*ATG16L2* rs10898880				
AA	20	101	1.00 (Reference)	
AC	64	171	1.97 (1.12–3.44)	**.018**
CC	34	78	2.29 (1.23–4.25)	**.009**
C versus A			1.51 (1.1–2.06)	**.010**

Note

*p* value in bold means statistically significant.

* Age, gender, BMI, smoking, drinking, family history of cancer, EBV‐DNA, chemotherapy, and TNM stage.

## 4 DISCUSSION

In present study, we investigated the associations of 10 potentially functional SNPs in *ATG2B, ATG10, ATG12*, and *ATG16L2* with the efficacy and toxicity of radiotherapy in 468 NPC patients. We found *ATG10* rs10514231, rs1864183, and rs4703533 were significantly associated with worse efficacy of radiotherapy at both at the primary tumor and lymph node, while *ATG16L2* rs10898880 was significantly associated with better efficacy of radiotherapy at both at the primary tumor and lymph node. Besides, we also found *ATG10* rs10514231 and *ATG16L2* rs10898880 were significantly associated with the occurrence of grade 3–4 oral mucositis and myelosuppression. These results suggest that potentially functional variants of ATGs might be useful biomarkers for predicting efficacy and toxicity of radiotherapy in NPC patients, once these results were validated by additional investigations.

With the rapid development of radio‐genomics, many studies have presented significant associations between genetic variants of candidate gene with the efficacy and toxicity of radiotherapy in NPC patients (Guo et al., 2017; Ma et al., 2017; Xie et al., 2014; Yu et al., 2016). Xie et al. (2014) found that the *p53* codon 72 polymorphism could be an independent prognostic marker for locoregionally advanced NPC. Guo et al. (2017) reported that *CDKN2A* rs3088440 was significantly related with a poorer treatment efficacy on the primary tumor and cervical lymph node after radiotherapy, and also with a decreased risk of grade 3–4 acute radiation‐induced myelosuppression. Ma et al. (2017) found that polymorphisms in angiogenesis related genes could contribute to clinical outcomes of radiotherapy in NPC patients. Yu et al. (2016) detected that *CTNNB1* rs1880481 and rs3864004, and *GSK3β* rs3755557 were significantly associated with poorer efficacy of radiotherapy in NPC patients, while *GSK3β* rs375557 and *APC* rs454886 were correlated with acute grade 3–4 radiation‐induced dermatitis and oral mucositis, respectively. These findings above revealed that genetic variants could potentially work as the indicator of efficacy and toxicity of radiotherapy in NPC patients.

Emerging evidence has revealed that autophagy process, which degrades intracellular components through the lysosomal machinery, plays an essential role in the process of cancer development and progression (Avalos et al., 2014; Mizushima, Levine, Cuervo, & Klionsky, 2008), while *ATGs* could control autophagic formation, and directly or indirectly accelerate cancer development and progression (Levine & Kroemer, 2008). Xie et al. (2016) identified that *ATG10* rs10514231, rs1864182 and rs1864183 were associated with poor lung cancer survival and positively correlated with *ATG10* expression. In current study, we also found *ATG10* rs10514231, rs1864183, and rs4703533 were significantly associated with worse efficacy of radiotherapy at both primary tumor and lymph node. Qin et al. (2013) reported that *ATG10* rs1864182 and rs10514231 were significantly associated with a decreased risk of breast cancer in Chinese population. Yuan et al. (2017) also revealed that genetic variations in *ATGs* were significantly associated with clinical outcomes of advanced lung adenocarcinoma treated with gefitinib. Recently, Wen et al. (2018) found *ATG16L2* rs10898880 contributed to a better prognosis of patients with non‐small cell lung cancer (NSCLC) after definitive radiotherapy, and a greater risk of developing severe radiation pneumonitis. This results were similar to the findings in current study, which revealed that *ATG16L2* rs10898880 was significantly associated with better efficacy of radiotherapy at both at the primary tumor and lymph node, and had a greater risk of developing grade 3–4 oral mucositis and myelosuppression.

## 5 CONCLUSIONS

Conclusively, we identified *ATG10* rs10514231, rs1864183, rs4703533, and *ATG16L2* rs10898880 could contribute to the efficacy and toxicity of radiotherapy in NPC patients. Further investigation of the underlying molecular mechanisms to explain how these polymorphisms affect response to radiotherapy and prospective clinical trials in NPC patients are needed to validate our results.

## CONFLICTS OF INTEREST

The authors declare that they have no conflict of interest.

## AUTHOR CONTRIBUTIONS

L.Z. and Y.Z. conceived and designed the experiments. L.Z. and Y.Z. performed the experiments. L.Z. and Y.Z. analyzed the data and wrote the paper.
